# Androgenic‐anabolic steroid abuse trend and management: A prospective, cross‐sectional, questionnaire‐based survey

**DOI:** 10.1002/hsr2.1032

**Published:** 2023-01-09

**Authors:** Manaf Al Hashimi, Yasser Farahat, Hussein Kandil, Ismail Al Khalidi

**Affiliations:** ^1^ Urology Department Burjeel Hospital Abu Dhabi UAE; ^2^ Khalifa University College of Medicine and Health Sciences Abu Dhabi UAE; ^3^ Urology Department Shaikh Khalifa Hospital Umm Al Quwain UAE; ^4^ Fakih IVF Fertility Center Abu Dhabi UAE; ^5^ Urology Department Al Garhoud Hospital Dubai UAE

**Keywords:** androgenic anabolic steroid, andrology, reproductive health, urology

## Abstract

**Background and Aims:**

Androgenic‐anabolic steroid (AAS) abuse is a global health concern, studies revealing an increasing trend of abuse and deleterious effects on reproductive health. Unfortunately, there is no consensus about management pathways due to the lack of specific guidelines.

**Methods:**

A prospective study, multicentre, online survey, composed of 30 questions, was conducted to investigate the current trend of AAS abuse and the management followed by practitioners from different specialities dealing with this condition.

**Results:**

A total of 151 respondents were included. The majority were general urologists (68.21%), andrologists (22.51%), and endocrinologists (9.28%). An increasing trend of AAS abuse was noticed by 90.73% of participants mostly in young age populations. Most of AAS abusers were presented with infertility (64.24%) and erectile dysfunction (59.60%), and their investigations showed abnormal semen analysis (77.48%), abnormal hormones (follicle‐stimulating hormone, luteinizing hormone, testosterone, and estradiol) (94.70%), and reduction in testicular size (50.33%). Most of respondents expected: the need of long duration for spontaneous recovery (6–12 months), relapse of AAS abuse in one‐third of patients, less knowledge about the adverse effects (39.74%), and risk of drug dependence (54.30%). Immediate treatment was the most offered plan of management (44.37%) followed by a waiting spontaneous recovery (32.45%), while the remaining would refer the patients to an either endocrinologist or andrologist. The treating physicians did not follow specific guidelines and most of participants (44.44%) reverted to their personal experience in the management.

**Conclusions:**

Our study revealed an increasing trend of AAS abuse, deleterious effects of AAS use on reproductive health, and lack of consensuses among the treating physicians regarding the management of related adverse effects. Our study could be considered a call to the scientific bodies to have more studies, establish guidelines for management, and to have better awareness of this serious public health concern.

## 1 INTRODUCTION

Currently, the use of performance‐enhancing drugs (PED), which are commonly used for enhancing muscular development and physical training as well as appearance in young and middle‐aged men, has increased in many societies and communities all over the globe.^1^, ^2^, ^3^, ^4^ These PED mainly contain androgens, known as androgenic anabolic steroids (AAS), which have negative effects on the hypothalamic–pituitary axis (HPA), leading to deterioration in testicular function that results in a unique condition known as anabolic steroid‐induced hypogonadism (ASIH). Eventually, this condition has a negative impact on sexual health and fertility; therefore, the abuse of these drugs has become a public health concern.^5^, ^6^, ^7^ The withdrawal of AAS results in sexual symptoms such as erectile dysfunction, loss of desire, impaired ejaculation, gynecomastia along with significant sperms abnormalities like oligospermia and azoospermia, which can manifest considerable fertility impairment.^8^, ^9^, ^10^ Discontinuing the use of AAS may result in spontaneous recovery of the associated negative effects; however, it may take several months to years and the effects may remain permanent in several patients.^11^, ^12^, ^13^

Due to a lack of publications comprising only a few relevant case series, very few large‐volume studies and paucity of information in the peer‐reviewed literatures describing the demographics, characteristics, and psychologic profile of AAS users, the understanding of the negative effects of AAS use and the development of a meta‐analyses to cover the scope of this matter has been hindered.^11^, ^12^, ^13^ Furthermore, there are no comprehensive management recommendations or guidelines available for the treatment of AAS‐induced complications such as infertility and ASIH. The therapeutic regimens available based on different studies may have shown some improvement in the condition; however, these studies are not controlled, and the medications used are off‐label.^14^, ^15^, ^16^ These facts further complicate the management of AAS‐abuse‐related adverse effects because there is no specific expected duration for spontaneous recovery. There are also no specific recommendations on what type or doses, or duration of medications can be used in the treatment. In addition, the proposed treatment itself could result in further deterioration and adverse effects.

All these shortages are real good proposals for future studies and were the basis of conducting our study. To the best of our knowledge, this is the first survey study ever to explore the current practice of urologists, andrologists, and endocrinologists involving in AAS management and document the prevalence, evaluation methods, and treatment modalities by determining the areas of the agreement and disagreement among the practitioners and to prepare a common guideline or recommendation. This study will be of value to all practitioners in this field and international scientific bodies to address the weak points in the current practice and improve the management pathways.

## 2 MATERIALS AND METHODS

An online survey was conducted to investigate the current trend of AAS abuse, and the management guidelines followed by practitioners from different specialists dealing with this condition (urologists, andrologists, and endocrinologists). It was a prospective, cross‐sectional, multicentre, observational survey, composed of 30 questions, which aimed to identify the responders background speciality, country, and practice setting (Q1–3), AAS abuse trend, age of abusers, purpose and advice of intake, the source of AAS pre‐use counselling, duration and frequency of the courses (Q4–9), the most common presentation symptoms, signs and investigations performed (Q10–16), the management guidelines and medications used (Q17–21), AAS users attitude, the current shortcomings in management guidelines, related educational activity and awareness programs (Q22–30).

We used the Google survey tool to formalize the survey. A link of the survey was sent to urologists, andrologists, and endocrinologists through LinkedIn, database emails of Arab Society of Urology, and other social media groups of concerned specialities in different countries. Only the fully attempted survey forms were included.

All statistical analysis was performed using the software STATA version 15.0. Each characteristic was illustrated appropriately. For continuous variables, data were formulated and presented using means ± SD. For categorical data, the number and percentages were used in the data summaries.

The study was approved by the ethical committee of Arab Society of Urology and all surveyors were asked in the first page of the survey to consent for using their data and responses for publication.

## 3 RESULTS

The survey link was sent to 370 specialists. A total of 171 responses were received, out of which 20 incomplete survey responses were excluded. Finally, a total of 151 responses were included. Out of 151 physicians, 146 (96.69%) were men and 5 (3.31%) were women. A total of 110 (72.84%) physicians belonged to private and 41 (27.16%) to governmental hospitals or clinics. In the study, 103 (68.21%) practitioners were general urologists, 34 (22.51%) andrologists, and 14 (9.28%) endocrinologists. Among the 19 countries from different continents, the UAE was the highest in representation (29.8%) followed by equal representation from Oman and Kuwait (9.27%), as depicted in Table 1.

**Table 1:** Summary statistics of survey responders character

Parameter	Overall (*N* = 151)
Age	
*N*	151
Mean	48.56
SD	9.84
Range (min, max)	(30.00, 70.00)
Gender, *n* (%)	
Male	146 (96.69)
Female	5 (3.31)
Clinical practice type, *n* (%)	
Government hospital	41 (27.16)
Private hospital	110 (72.84)
Practice speciality, *n* (%)	
Andrologist	34 (22.51)
Endocrinologist	14 (9.28)
General urologist	103 (68.21)
Clinical practice country, *n* (%)	
Bahrain	13 (8.61)
Egypt	10 (6.62)
France	1 (0.66)
India	10 (6.62)
Iraq	10 (6.62)
Ireland	1 (0.66)
Kuwait	14 (9.29)
Malaysia	1 (0.66)
Morocco	1 (0.66)
Mauritania	1 (0.66)
North Macedonia	1 (0.66)
Oman	14 (9.29)
Pakistan	2 (1.32)
Qatar	10 (6.62)
Saudi Arabia	10 (6.62)
Sweden	1 (0.66)
Tunisia	1 (0.66)
UAE	45 (29.80)
UK	5 (3.31)

The majority (90.73%) of the physicians noticed an increasing trend of AAS abuse and estimated the increase to be moderate (56.95%), and less commonly observed as mild (37.09%), and severe (5.96%). The respondents stated the most common age categories of AAS users are 20–30 years (74.83%), 30–40 years (13.25%), above 50 years (8.61%), and 40–50 years (3.31%). The purposes of AAS utilization among users were to enhance their fitness for training (70.2%) and physical appearance (60.26%), while other reasons (10.6%) to improve self‐esteem or body image. The sources of patient counseling before utilization of AAS were the physical trainer (the coach) (70.2%), followed by advice from friends (56.95%), social media (53.64%), and self‐request (38.41%). The duration of AAS abuse among most of them was between 1 and 3 years (47.68%) followed by less than 1 year (39.74%) and 4–6 years (12.58%). The observed duration of each course was 4–8 weeks (50.99%), followed by 1–4 weeks (16.56%), 8–12 weeks (23.18%), and more than 12 weeks (9.27%), while receiving two courses per year by AAS users was the most common form of use (53.64%), followed by one course per year (25.17%), three courses per year (8.61%), and more than three courses per year (12.58%). Most of the AAS abusers (47.02%) mentioned consumption after being proactively asked for it, (41.72%) of them mentioned it at presentation, and the others (10.72%) remained discrete and released information after receiving abnormal laboratory results.

Regarding the presenting symptoms, infertility was the most common (64.24%) followed by erectile dysfunction (59.60%) and loss of sexual desire (56.95%). For the evaluation of AAS users, the hormonal profile was the most performed initial investigation (90.07%), followed by semen analysis (72.19%). The results of evaluation revealed low testosterone (69.54%), low luteinizing hormone (LH) (58.28%), low follicle‐stimulating hormone (FSH) (41.06%), and high estradiol levels (23.18%). More than three‐quarters of the participants (77.48%) found abnormal semen parameters of AAS users at presentation, while the remaining (22.52%) claimed it as being normal. On clinical evaluation of the AAS users, testicular size reduction was perceived by half the participants (50.33%), testicular atrophy by 3.97%, and 45.70% noted no change in testicular size, as shown in Table 2.

**Table 2:** Summary statistics of patients' findings on evaluation

Parameter	Overall (*N* = 151)
Clinical presentations, *n* (%)	
Ejaculatory dysfunction	14 (9.27)
Erectile dysfunction	90 (59.60)
Gynecomastia	51 (33.77)
Infertility	97 (64.24)
Loss of sexual desire	86 (56.95)
Most of above	3 (1.99)
Orgasmic dysfunction	19 (12.58)
Evaluate AAS users, *n* (%)	
CBC	77 (50.99)
Hormonal profile	136 (90.07)
Liver function tests	75 (49.67)
Scrotal ultrasound	44 (29.14)
Semen analysis	109 (72.19)
Others	14 (9.27)
Hormonal assessment, *n* (%)	
Any hormonal abnormalities	143 (94.70)
High estradiol	35 (23.18)
Low FSH	62 (41.06)
Low LH	85 (56.29)
Low testosterone	105 (69.54)
Semen analysis, *n* (%)	
I don't request semen analysis	15 (9.93)
It is commonly abnormal	117 (77.48)
It is commonly normal	19 (12.58)
Abnormality of semen analysis, *n* (%)	
Asthnospermia	32 (21.19)
Azoospermia	22 (14.57)
Combination of the above	62 (41.06)
Oligospermia	90 (59.60)
Teratospermia	8 (5.30)
Size of tests in AAS users, *n* (%)	
Atrophy of testes	6 (3.97)
Normal size testes	69 (45.70)
Smaller size testes	76 (50.33)

**Figure 1:**
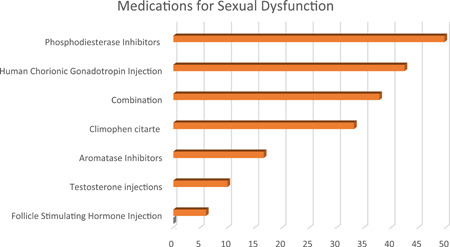
Showing the percentages of different medications used for treating androgenic‐anabolic steroid abuse‐related sexual dysfunction

**Figure 2:**
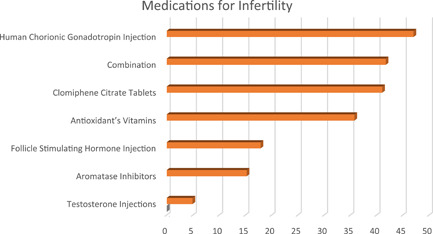
Showing the percentages of different medications used for treating androgenic‐anabolic steroid abuse‐related infertility

Regarding the management of AAS‐related adverse effects showed that the immediate treatment was the most offered plan of management (44.37%) followed by a waiting for spontaneous recovery (32.45%) and referring the patient to an endocrinologist (13.91%), or an andrologist (9.27%). Spontaneous recovery of sexual symptoms following the termination of AAS use was mostly believed to take 6–12 months (42.38%), followed by 3–6 months (32.45%), more than a year (17.88%), and 1–3 months (7.29%). Most prescribed medications to treat sexual dysfunction were phosphodiesterase inhibitors (49.69%), followed by human chorionic gonadotropin (hCG) injection (42.38%), combination of these medications (37.75%), clomiphene citrate (CC) (33.11%), aromatase inhibitors (16.56%), testosterone injections (9.90%), and FSH injections (5.96%); for the infertility, hCG was the most used therapy (47.02%), followed by combination of medications (41.72%), CC (41.06%), antioxidants (35.76%), FSH injections (17.88%), aromatase inhibitors (15.23%), and testosterone injections (4.90%) as in Figures 1 and 2.

Gynecomastia was encountered as rare presentation by 51.66% of participants, was commonly observed in 35.10%, very common in 7.95%, and not examined in 5.29% of participants. The offered management was awaiting spontaneous recovery is more commonly practiced (49.10%), followed by using tamoxifen (29.8%), anastrazole (17.22%), and Letrizole (3.88%). A total of two‐third of participants (65.56%) suggested sperms cryopreservation for AAS abusers, especially for those presenting with infertility and the remaining (34.44%) were not suggesting.

The assessment of the AAS abusers' knowledge about related adverse effects revealed they were less knowledgeable (39.74%), knowledgeable (31.13%), had misconceptions (23.18%), or were very knowledgeable (5.95%). Most of the respondents (68) expected relapse of AAS use in 60%–90% of patients, followed by (54) in 30%–60% of patients, (20) in 10%–30% of patients, and (9) in less than 10% of their patients.

Along the course of management, AAS users were perceived by their care providers as either weakly (41.37%) or strictly (38.05%) compliant to the prescribed treatment, while 10.60% agreed that they were hesitant and 9.98% not convinced to start the medications. The notion of AAS‐related drug dependence was acknowledged by most of the participants (54.30%), while the remaining (45.70%) did not believe so.

Regarding the concepts of treatment of AAS‐related adverse effects, most of participants (44.44%) reverted to their personal experience, while 33.77% followed the insights from relevant studies, and 21.79% followed treatment guidelines of other types of hormonal abnormalities (not specific to AAS abuse). In addition, most of the participants (95.60%) denied participation in any related scientific activity while the remaining (4.40%) rarely attended. Almost all the respondents reported the lack of the national awareness program about the AAS abuse in their countries. Finally, an endocrinologist was the most chosen healthcare provider identified by the participants to manage AAS abuse adverse effects (40.93%) followed by an andrologist (31.79%), a general urologist (22.98%), and by a team of different specialities (4.30%).

## 4 DISCUSSION

Many studies concluded the findings of significant increasing trend of AAS abuse in young populations, not only to enhance physical training but also physical appearance. This finding is confirmed in our survey study as most of respondents (90%) stated the increasing trend of AAS abuse among their patients, which is consistent with other studies both in this region as well as globally.^3^, ^4^, ^5^, ^17^, ^18^, ^19^ AAS preparations are classified as Schedule III Controlled Substances in the United States and in many other regions, and it is considered illegal to pose AAS without prescription,^3^ but still are widely used, indicating the lack of awareness and control over testosterone prescriptions.

AAS abusers' attitudes were explored in our study, including their knowledge about the adverse effects of AAS abuse on sexual and fertility health as most of respondents believed their patients are less knowledgeable or had misconceptions about the AAS adverse effects. This finding also was acknowledged by Ahmed  et al. study^20^ in Jeddah/Saudi Arabia Kingdom who concluded that half of included patients exhibited poor knowledge regarding the side effects of AASs. These are alarming factors, proving the underestimation of AAS adverse effects, thereby raising the urgent need of national awareness programs to provide the knowledge and explanation of adverse effects of AAS abuse and to have better‐governed control over dispensing these medications.

The negative impact of AAS abuse on HPA and related induced hypogonadism is well‐recognized adverse effects and eventual sexual dysfunction and impairment of fertility. Although the effects are variable according to the type of used preparations, doses, and duration, most of studies^8^, ^9^, ^10^, ^11^, ^12^, ^13^ showed significant deleterious effects and need of treatment to override these adverse effects. These findings are confirmed in our study, as the most common presentations were infertility and erectile dysfunction, and the most common findings were abnormal hormones and semen analysis. Most of the respondents expected a long waiting time (6–12 months) for spontaneous recovery of these adverse effects which could be temporary or sometimes permanent as noticed in other studies,^12^, ^13^, ^21^ and expected relapse of AAS use in 60%–90% of their patients, which could raise the risk of permanent adverse effects as more commonly associated with prolonged and relapsed AAS courses.

Another important finding is half of respondents in our study noted AAS use dependence in their patients, which is considered another serious negative impact and community concern and was noticed in other studies like Kanayama G study^22^ which rated its incidence as 30% in their cohorts. Furthermore, Horwitz et al.^23^ concluded that AAS users have an increased risk of dying and significantly more hospital admissions than their nonuser peers. These remarkable findings raised the public health concerns about endangering the younger population health and warn the concerned authorities to make more rules and policies to contain the abuse of AAS.

Regarding the management of AAS abuse adverse effects, our study revealed no consensus among the practitioners, as 41% offered immediate treatment while 32% preferred waiting for spontaneous recovery. Also, there is no consensus on the type and duration of medications for the treatment of AAS abuse‐related infertility or sexual symptoms. The same findings were elicited in other studies^14^, ^15^, ^16^, ^24^, ^25^, ^26^ with different recommendations about the pathways and medications. The options are: (i) immediate discontinuation with no medical therapy; (ii) discontinuation and initiation of a limited course of clomiphene therapy; (iii) discontinuation and initiation of a limited course of hCG therapy; or (iv) conversion of non‐prescription AASs to prescription testosterone. As the US Food and Drug Administration has not approved the use of clomiphene, hCG, or testosterone for the treatment of AAS withdrawal, and there are no clinical trials of medical therapy for AAS withdrawal, so all the suggested medications are off label, a fact adds more controversies on the management of this condition. Tatem et al.^14^ provide a pathway of treatment of AAS adverse effects through immediate stopping the AAS intake followed by 3 months course of hCG + CC and re‐evaluation after, while Al Hashimi^26^ showed another reliable pathway of treatment with initial 3 months waiting for spontaneous recovery, if not responding then to start hCG + CC, and re‐evaluation every month with possible titration of doses to avoid over treatment and possible more side effects, also his study addressed the treatment of associated high estradiol hormones and gynecomastia, which highly contributed to the adverse effects of AAS abuse.

The lack of conclusive and well‐performed studies prevents the possibility of giving clear recommendations on how to manage AAS abuse and its consequences especially infertility and sexual dysfunction, this could explain the diversity of practitioner's decisions about the treatment, as most of participants depends on their personal experience and about one‐third of them follow the insights from studies related to this topic. The lack of focus from andrology societies on this subject and their failure to organize related activities, and motivate the practitioners to engage in such events, could explain that three‐fourth of the participants were not attending any related scientific activity or to have national awareness program. All these factors raise the debates about management of the condition and increase its burden on public health.

## 5 CONCLUSIONS

Our study concluded an increasing trend of AAS abuse in different regions all over the globe, which imposes deleterious effects on sexual and fertility health. Furthermore, there is a lack of consensus regarding the management of related adverse effects and follow‐up plans among the treating physicians and lack of studies and academic activities to address the problem. Therefore, it is important to conduct further studies and generate specific guidelines for the management of this condition.

## AUTHOR CONTRIBUTIONS

**Manaf Al Hashimi**: Conceptualization; data curation; formal analysis; investigation; methodology; project administration; resources; supervision; validation; visualization; writing – original draft; writing – review & editing. **Yasser Farahat**: Resources; validation. **Hussein Kandil**: Writing – review & editing. **Ismail Al Khalidi**: Writing – review & editing.

## CONFLICT OF INTEREST

The authors declare no conflict of interest.

## TRANSPARENCY STATEMENT

The lead author Manaf Al Hashimi affirms that this manuscript is an honest, accurate, and transparent account of the study being reported; that no important aspects of the study have been omitted; and that any discrepancies from the study as planned (and, if relevant, registered) have been explained.

## Data Availability

All authors have read and approved the final version of the manuscript (Dr. Manaf Al Hashimi) had full access to all the data in this study and take complete responsibility for the integrity of the data and the accuracy of the data analysis. Herewith, the authors confirm there are no retracted references. The data that support the findings of this study are available on request from the corresponding author. The data are not publicly available due to privacy or ethical restrictions.
